# Electron Dynamics and Correlations During High-Order Harmonic Generation in Be

**DOI:** 10.3389/fchem.2022.809137

**Published:** 2022-01-31

**Authors:** Eric Kutscher, Anton N. Artemyev, Philipp V. Demekhin

**Affiliations:** Institut für Physik und CINSaT, Universität Kassel, Kassel, Germany

**Keywords:** light-matter interaction, strong-field ionisation, high-harmonic generation, electron correlations, restricted-active-space, theoretical and numerical methods

## Abstract

We investigate theoretically the high-order harmonic generation in beryllium atom irradiated by a short 1850 nm linearly polarized laser pulse in the intermediate strong-field ionization regime with the Keldysh parameter of 0.85. To this end, the respective time-dependent Schrödinger equation is solved by the time-dependent restricted-active-space configuration-interaction (TD-RASCI) method. By systematically increasing the active space of included configurations, we demonstrate an individual effect of different physical processes evoked by the pulse, which, all together, significantly enrich and extend the computed high-order harmonic generation spectrum.

## Introduction

The high-order harmonic generation (HHG (Corkum, 1993)) is one of the most fascinating processes arising due to the nonlinear response of matter to strong laser pulses. It gave rise to a new efficient way of generating high-frequency XUV laser pulses (Brabec and Krausz, 2000) and opened up a door to the area of attosecond physics (Kienberger et al., 2004). Many efforts have been spent to provide a detailed theoretical explanation of the HHG processes. Already the semi-classical three-step model (Lewenstein et al., 1994) explains the well-known cutoff law (Krause et al., 1992) in the harmonic spectra, as is confirmed by numerous successful calculations of HHG spectra performed within the single-active electron (SAE) approximation (Bandrauk et al., 2009; Frolov et al., 2009; Ivanov and Kheifets, 2009; Le et al., 2009; Han and Madsen, 2010; Chelkowski et al., 2012; Fu et al., 2017). Going beyond the SAE approximation and solving fully the many-body time-dependent Schrödinger equation (TDSE) is a formidable computational task. Nevertheless, several attempts to study dynamics and correlations of inactive electrons have been performed (Gordon et al., 2006; Miyagi and Madsen, 2013; Sato et al., 2016; Artemyev et al., 2017a; Tikhomirov et al., 2017) by either reducing dimensionality of the problem or simplifying many-body interactions. More details on the HHG phenomenon and key concepts of the strong-field attosecond science can be found, e.g., in the review article (Krausz and Ivanov, 2009).

In order to accurately describe the three-dimensional four-electron beryllium atom exposed to a strong infrared laser pulse, which is a subject of the present theoretical study, several approaches were already reported. For instance, a model approach of Ref. (NgokoDjiokap and Starace, 2013). introduces an effective potential which replaces the two innermost 1*s* ^2^ electrons. The HHG spectra obtained in the multiphoton regime exhibit a strong resonant enhancement due to doubly-excited intermediate autoionizing states. The two accurate approaches designed to describe the full dimensional correlated dynamics of the electrons are the well-known time-dependent configuration-interaction (TDCI (Klamroth, 2003; Krause et al., 2005)) and multiconfigurational time-dependent Hartree-Fock (MCTDHF (Zanghellini et al., 2003; Kato and Kono, 2004; Nest et al., 2005; Alon et al., 2007; Haxton et al., 2011; Hochstuhl and Bonitz, 2011)) methods. In the former approach, the many-electron wave function is expanded over a basis of chosen configurations described by an optimized Slater determinant with a time-dependent expansion coefficient. In the latter, the basis functions are time-dependent in addition and optimized at each time step. Main concepts of the time-dependent formulation of the computational methods can be found, e.g., in the book (Meyer et al., 2009).

Unfortunately, computational efforts of TDCI and MCTDHF methods increase very rapidly with the number of included basis states. Therefore, more efficient methods, such as time-dependent complete-active-space self-consistent-field theory (TD-CASSCF (Sato et al., 2016; Tikhomirov et al., 2017)), the time-dependent restricted-active-space self-consistent-field theory (TD-RASSCF (Miyagi and Madsen, 2013)), and the time-dependent two-particle reduced-density-matrix theory (TD-2RDM (Lackner et al., 2017)), were used to study HHG spectra in beryllium atom. All HHG spectra of Be, computed by these methods beyond the SAE approximation, demonstrate a significance of electron dynamics and correlations in the generation of high-order harmonic spectra. While the HHG spectra obtained in Refs. (Sato et al., 2016; Lackner et al., 2017). on three-dimensional beryllium atom are most accurate and yield reliable predictions, it is rather difficult to extract from those calculations individual impacts of important physical effects on HHG spectra in a transparent way.

In the present work, we aim at revealing those physical effects. To this end, we employ the time-dependent restricted-active-space configuration-interaction (TD-RASCI (Hochstuhl and Bonitz, 2012)) method, adopted in our previous works (Artemyev et al., 2016; Artemyev et al., 2017a; Artemyev et al., 2017b; Artemyev et al., 2019) to study interaction of two electrons of helium atom with intense laser pulses. In this method, a preselected optimized set of single-particle orbitals is used as a basis for the time-independent Slater determinants and a chosen number of electrons is allowed to occupy excited and continuum single-particle states, where the latter are sought as the time-dependent wave packets. By systematically enhancing the active space and thus enabling specific physical processes step by step, one can differentiate individual contributions to the electron dynamics and correlation, and can thus explore the physical role of relevant processes and their impact on the HHG. For the present needs, we extend our realization of the TD-RASCI method to study dynamics and correlations of arbitrary number of electrons.

## Theory

The present realization of the TD-RASCI method was introduced in detail in our previous works on He (Artemyev et al., 2016; Artemyev et al., 2017a; Artemyev et al., 2017b). Therefore, only developments of the method which are necessary to describe field-driven electron dynamics in Be are discussed below.

We describe the light-matter interaction in the velocity gauge, where a fast convergence over the angular momentum of photoelectron wave packets is inherent (Cormier and Lambropoulos, 1996). Thereby, in the electric dipole approximation, the total Hamiltonian $\hat{H}$ of Be atom interacting with a linearly polarized laser pulse reads (atomic units are used throughout this paper)

$$\hat{H}=\overset{4}{\underset{j=1}{\sum }}\left(-\frac{1}{2}{\vec{\nabla }}_{j}^{2}-\frac{4}{r_{j}}-i\nabla_{z_{j}}A_{0}g\left(t\right)\sin\left(\omega t\right)\right)+\underset{i<j}{\sum }\frac{1}{\left|{\vec{r}}_{i}-{\vec{r}}_{j}\right|}\;.$$ (1)

Here, the first sum accounts for kinetic energy, potential energy of the nuclear-electron interaction, and light-matter interaction for all electrons, while the second one describes the Coulomb repulsion between electrons. The vector potential of the pulse is described by its carrier frequency *ω*, time envelope *g*(*t*) and peak amplitude *A*_0_, which is related to the peak intensity *I*_0_ via $I_{0}=A_{0}^{2}\frac{\omega^{2}}{8\pi \alpha }$ (*α* ≈ 1/137.036 is the fine-structure constant).

The present calculations were performed for illustrative laser pulses with peak intensity *I*_0_ = 2 × 10^13^ W/cm^2^ and wavelength *λ* = 1850 nm. The corresponding Keldysh (Keldysh, 1965) parameter *γ* = 0.85 indicates that the strong-field ionization of Be takes place in the intermediate regime between tunnel and multiphoton ionization, and far from a barrier suppression regime. With such a choice of the pulse parameters, the probability of double-ionization of Be atom is low, as compared to that of its single-ionization. Thereby, one can neglect any double-ionization of Be atom and assume that only one of its four electrons can populate continuum states. Nevertheless, electron dynamics in the ionic core (i.e., excitation caused by electron correlations or induced by interaction with the pulse) are not negligible and need to be considered. Therefore, we make the following ansatz for the total four-electron wave function of Be:

$$\Psi \left({\vec{r}}_{1},{\vec{r}}_{2},{\vec{r}}_{3},{\vec{r}}_{4},t\right)=\underset{ijkl}{\sum }a_{ijkl}\left(t\right)|\phi_{i}\phi_{j}\phi_{k}\phi_{l}\rangle +\underset{pqrs}{\sum }|\phi_{p}\phi_{q}\phi_{r}\psi_{s}\left(t\right)\rangle .$$ (2)

In the ansatz (2), two kinds of contributions in the total wave function are explicitly separated. The first sum over the |*ϕ*_*i*_ *ϕ*_*j*_ *ϕ*_*k*_ *ϕ*_*l*_⟩ configurations with the time-dependent expansion coefficients *a*_*ijkl*_(*t*) contains only preselected bound one-particle orbitals *ϕ*, while the second sum over |*ϕ*_*p*_ *ϕ*_*q*_ *ϕ*_*r*_ *ψ*_*s*_(*t*)⟩ configurations contains three bound orbitals *ϕ* and one photoelectron wave packet *ψ*(*t*), which is either excited beyond the basis of preselected bound orbitals *ϕ* or belongs to continuum states. Configurations of both kinds are given by linear combinations of Slater determinants constructed following angular momentum summation rules. The two one-particle basis sets {*ϕ*_*α*_ ≡ *ϕ*_*nℓm*_} and {*ψ*_*s*_(*t*) ≡ *ψ*_*ɛℓm*_(*t*)} are mutually orthogonal (Artemyev et al., 2016). Both summations in Eq. 2 run over all possible combinations which can be formed within the selected basis sets. In order to describe one-electron orbitals, we use the finite-element discrete-variable representation (FEDVR) scheme (Manolopoulos and Wyatt, 1988; Rescigno and McCurdy, 2000; McCurdy et al., 2004; Demekhin et al., 2013; Artemyev et al., 2015). Thereby, the radial coordinate of the three-dimensional basis element $\xi_{\lambda }\left(\vec{r}\;\right)$ is represented in the basis set of the normalized Lagrange polynomials, which are constructed over a Gauss-Lobatto grid. The normalized stationary orbitals $\phi_{\alpha }=\sum_{\lambda }c_{\lambda }^{\;\alpha }\;\xi_{\lambda }$ and the time-dependent wave packets $\psi_{s}\left(t\right)=\sum_{\lambda }b_{\lambda }^{\;s}\left(t\right)\xi_{\lambda }$ are expanded with respect to the FEDVR basis elements *ξ*_*λ*_. The matrix elements containing one- and two-particle contributions can then be identified using symmetry properties and selection rules, and their analytical expressions can be found in Refs. (McCurdy et al., 2004; Artemyev et al., 2016; Artemyev et al., 2017a).

Within the TD-RASCI method, the full configuration-interaction character of the TDCI method is relaxed by allowing only a selected set of bound states to be included into the basis (Artemyev et al., 2016; Artemyev et al., 2017a; Artemyev et al., 2017b). In the case of Be atom, the method is also designed to choose a type of configurations which can be built out of a selected basis, of course, in a complete manner. In this way, we are able to study different levels of approximations and their influence on HHG spectrum, catching thereby the essential physics of the problem without losing accuracy. The simplest case is the SAE approximation, where three electrons are fixed in the configuration 1*s* ^2^ 2*s* and only one of the electrons is allowed to participate in the dynamics. More complex calculations incorporate a frozen-core approximation which keeps two electrons in the 1*s* ^2^ state, and thus single and double excitations of the two outermost electrons are allowed (hereafter referred to as 2FC). In beryllium, one can also consider further excitations of 1*s* and 2*s* electrons, which will be referred to as 2DC (dynamical-core) approximation. Depending on the allowed type of included configurations, this gives rise to the additional 1*s* ^2^ ionization or 2*s* ^2^ relaxation. In the former case, the active space included additional configurations with two electrons kept frozen in the 2*s* ^2^ state, whereas in the latter case, additional 3*s* ^2^ excitations in the presence of all former effects were allowed. The last step in complexity included in this study is the 1FC approximation. Here, only one electron is kept frozen in the 1*s* orbital, and all possible triple excitations are considered. This allows one to study the influence of three-electron dynamics and correlations on the HHG spectra. We stress again that in all considered approximations, the condition of a singly-occupied continuum is fulfilled. Summarizing, starting from SAE approximation, we consider the mentioned physical effects by going to 2FC, further to 2DC, and finally to 1FC approximations.

Time evolution of the total wave function (2) is given by the vector of the time-dependent expansion coefficients $\vec{B}\left(t\right)=\left\{a_{ijkl}\left(t\right);b_{\lambda }^{\;s}\left(t\right)\right\}$ . $\vec{B}\left(t\right)$ was propagated according to Hamiltonian (1). The propagation was carried out by the short-iterative Lanczos method (Park and Light, 1986). The initial ground state $\vec{B}\left(t=0\right)$ was obtained by the propagation in imaginary time (relaxation) in the absence of the external field. Finally, the HHG spectrum *I*(*ω*) was computed as the squared modulus of the Fourier-transformed acceleration of the total electric dipole moment as

$$I\left(\omega \right)=\frac{1}{{\left(2\pi \right)}^{1/2}}{\left|\int \frac{d^{2}D\left(t\right)}{dt^{2}}e^{-i\omega t}dt\right|}^{2},$$ (3)

with *D*(*t*) given by

$$D\left(t\right)=\left\langle \Psi \left({\vec{r}}_{1},{\vec{r}}_{2},{\vec{r}}_{3},{\vec{r}}_{4},t\right)|\overset{4}{\underset{i=1}{\sum }}{\vec{r}}_{i}|\Psi \left({\vec{r}}_{1},{\vec{r}}_{2},{\vec{r}}_{3},{\vec{r}}_{4},t\right)\right\rangle .$$ (4)

The present study was conducted for a trapezoidal pulse with a linearly growing front edge, a constant plateau with unit height, and a linearly falling back edge, each supporting 4 optical cycles. Thereby, an asymmetry due to the carrier-envelope phase can be neglected. The propagation was therefore performed in the time interval of [0, *T*_*f*_] with *T*_*f*_ ≈ 74 fs. The size of the radial box was chosen to be *R* *_max_* = 4500 a.u. The radial interval [0, *R*_max_] was divided into 2250 equidistant finite elements of 2 a.u. size, each covered by 10 Gauss-Lobatto points. The photoelectron wave packets {*ψ*_*s*_(*t*)} were described by the partial harmonics with *ℓ* ≤ 50. Because the photoelectron wave packet is initially localized within a relatively small box and its size gradually increases with time according to the TDSE, we could save computation efforts by incorporating a so-called “running grid.” To this end, the outermost wave-packet-free region of the grid with *r* > *R*_*cut*_(*t*) was neglected during the propagation. In this region, each harmonics of the wave packet must be smaller than a predefined accuracy parameter *ϵ* = 10^−10^, and the dynamical edge of this region *R*_*cut*_(*t*) must be updated at each time step. Finally, when *R*_max_ was reached, the wave packets are multiplied at each time step by a mask function (Artemyev et al., 2017a) to avoid any reflection from the boundary.

## Results and Discussion

**FIGURE 1:**
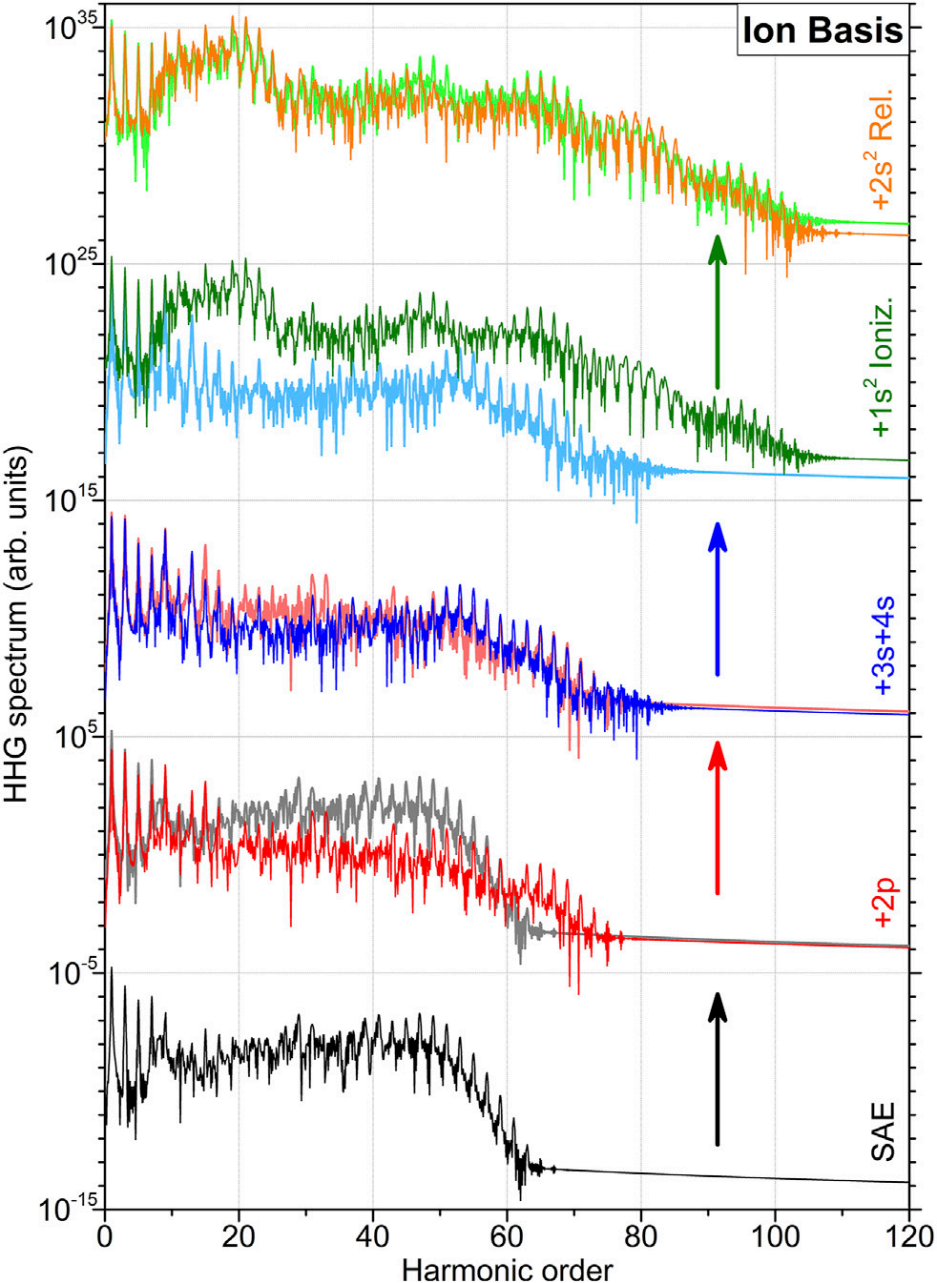
High-order harmonic generation spectra of beryllium atom computed in the basis set {*ϕ*_*α*_} of ionic orbitals. The calculations are performed in a systematic series of improving approximations used to incorporate dynamics of the bound electrons (indicated at the right-hand vertical axis). For a better comparison, the spectra in each row are compared with the ones calculated in the previous level of approximation (as indicated by the colored arrows) and are vertically shifted upwards by multiplying successively with 10^10^ starting with the lowermost spectrum, obtained in the SAE approximation. The next two spectra (from bottom to top) are obtained within the 2FC approximation, by sequentially extending the basis set of discrete orbitals $\left\{n\ell_{+}\right\}$ , first with 2*p*_+_ and then additionally with 3*s*_+_ and 4*s*_+_. The spectra shown in the two uppermost rows represent results obtained within the 2DC approximation, by additionally allowing first $1s_{+}^{2}$ ionization and then $2s_{+}^{2}$ relaxation.

**FIGURE 2:**
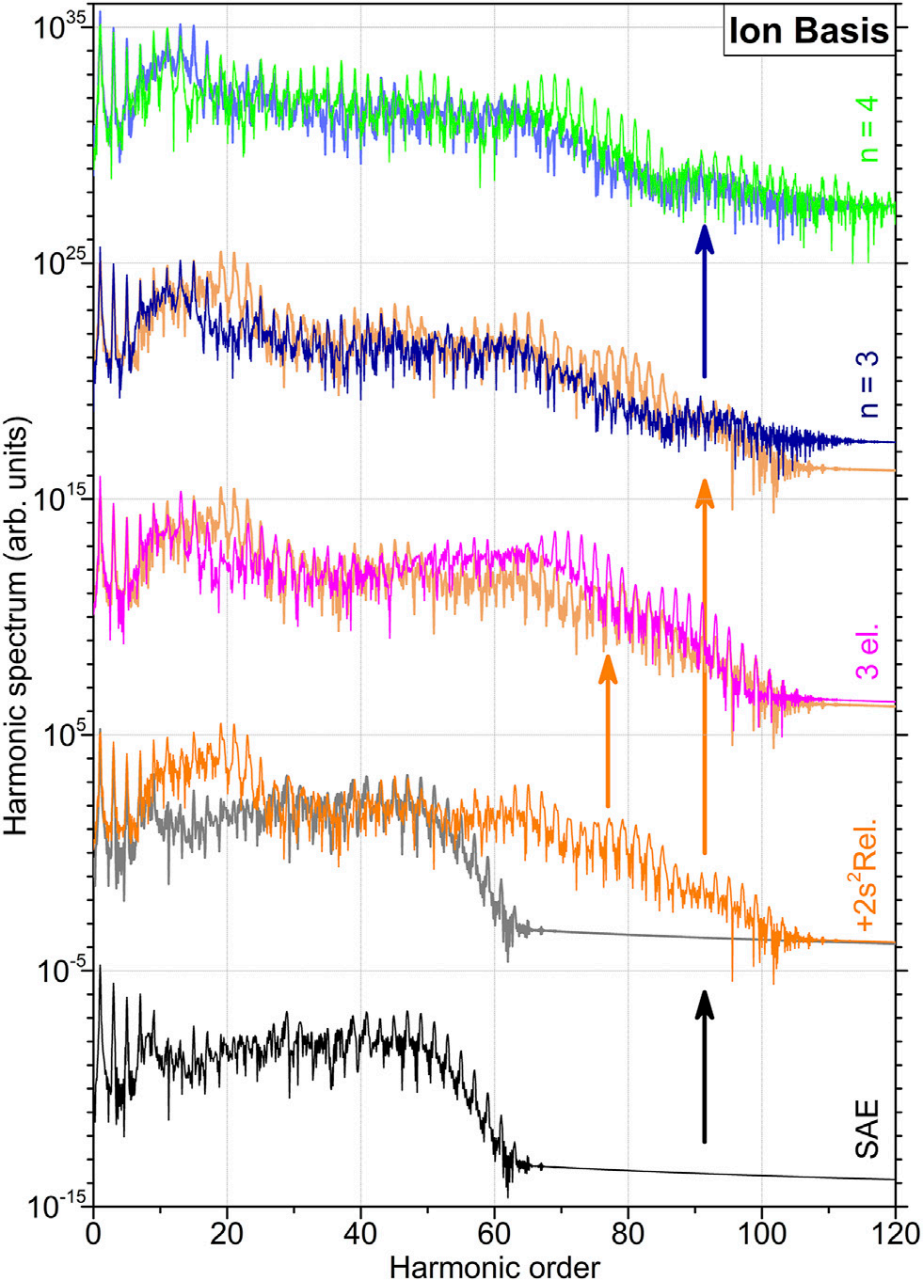
High-order harmonic generation spectra of beryllium atom computed in the basis set {*ϕ*_*α*_} of ionic states at higher levels of approximations (indicated at the right-hand vertical axis, see also caption of Figure 1 for details on the data representation). The two lowermost spectra, obtained in the SAE approximation and in the 2DC approximation by including physical effects up to $2s_{+}^{2}$ relaxation, are the same as the respective spectra in Figure 1. The spectrum in the middle, obtained in the 1FC approximation, illustrates an impact of the three-electron correlations, as compared to that of the 2DC approximation (also indicated by the respective colored arrow). The spectra shown in the two uppermost rows represent results obtained within the 2DC approximation by additionally (as compared to that of the 2DC approximation, see also respective colored arrows) extending the ionic basis set {*ϕ*_*α*_} first with all *nℓ*_+_ orbitals with principal quantum *n* = 3 and then with *n* = 4.

**FIGURE 3:**
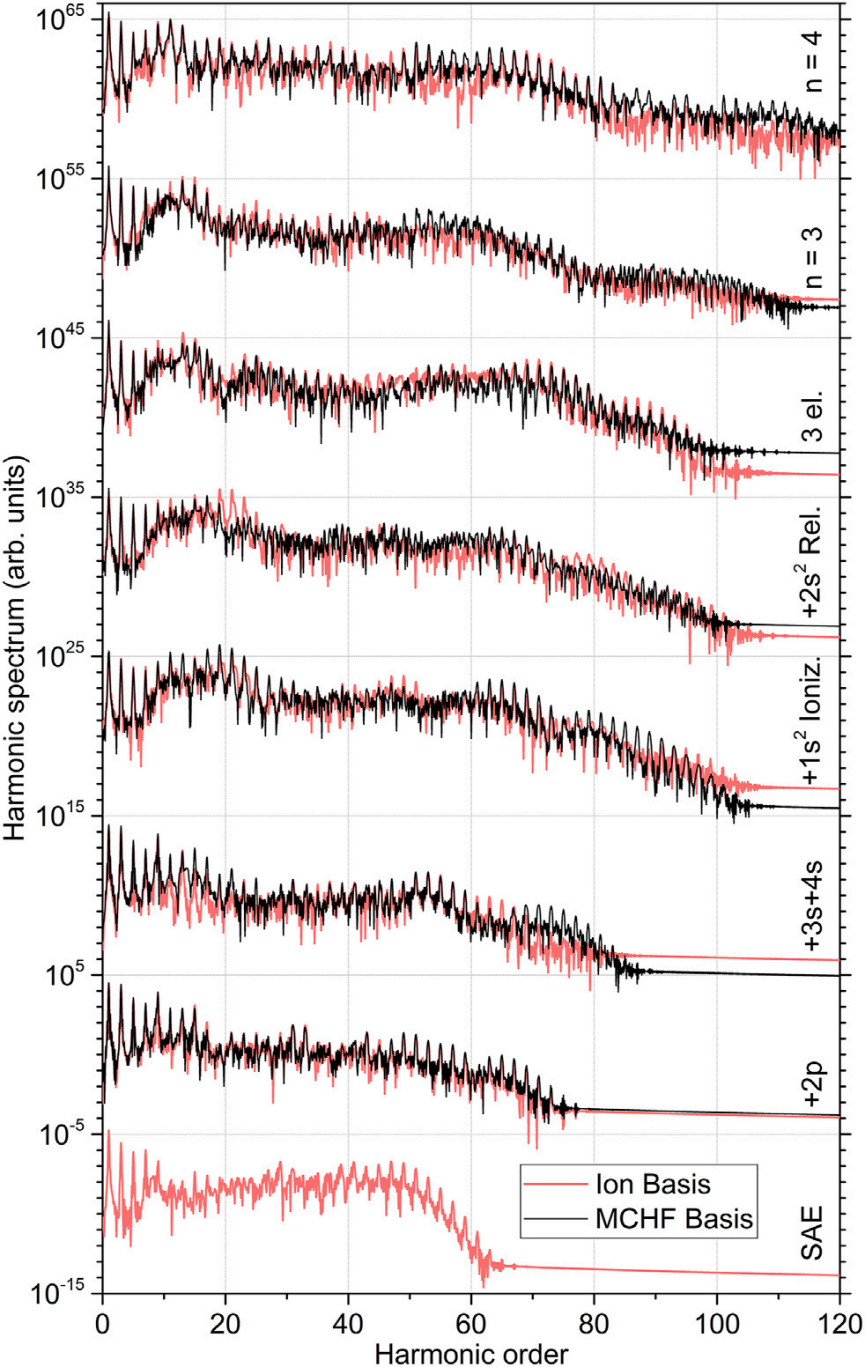
Comparison of the high-order harmonic generation spectra of beryllium atom computed using the ionic and MCHF basis sets in different level of systematically improving approximations (indicated at the right-hand vertical axis, see also captions of Figures 1, 2 for details on the data representation). The five lowermost spectra obtained in the ionic basis set correspond to those depicted Figure 1, while the three uppermost ones to the three uppermost spectra shown in Figure 2.

In the present calculations, we used two different basis sets for the description of the bound orbitals {*ϕ*_*α*_}. The first one is composed of the Hartree-Fock (HF) orbitals obtained by solving the stationary Schrödinger equation of the singly charged beryllium ion in the configuration of $1s_{+}^{2}2s_{+}^{1}$ (hereafter referred to as {*nℓ*_+_} ion basis set). This choice is natural for an accurate description of the final ionic state of the system. Here, we use all ionic orbitals with *n* ≤ 4 and *ℓ* ≤ 3 (i.e., up to 4*f*_+_ orbital). The initial neutral ground state of beryllium atom is built mainly from two configurations 1*s* ^2^ 2*s* ^2^ and 1*s* ^2^ 2*p* ^2^ (Fischer and Jönsson, 1994) and, thus, is accurately described by the multiconfigurational Hartree-Fock (MCHF) method. As the second basis set of the bound orbitals {*ϕ*_*α*_}, we used the minimal MCHF basis containing {1*s*, 2*s*, 2*p*} orbitals. Here, in order to describe the whole ionization process accurately, we extended it successively by adding all ionic orbitals *nℓ*_+_ and implying mutual orthogonalisation. In what follows, the HHG spectra obtained with the ionic basis set are depicted in Figures 1, 2, while comparison between the spectra obtained in different basis sets is shown in Figure 3. Figure 1 illustrates results of the lower-level 2FC and 2DC calculations, as obtained in the basis set with all ionic orbitals with the principal quantum number *n* = 2. In Figure 2, higher-level 2DC (with *n* = 3 and *n* = 4) and 1FC (3el.) calculations are compared with the best lower-level 2DC result.

The simplest calculations in the SAE approximation can be performed only in the ionic basis set of {1*s*_+_, 2*s*_+_} oribitals. Here, we allow one of the 2*s*_+_ electrons to be ionized, but include direct and exchange interactions between all bound and continuum electrons. The respective HHG spectrum is shown in the lowermost row of Figure 1. This spectrum exhibits a plateau of harmonics up to an order of about 50 and a sharp cutoff up to harmonic order of about 60. These results agree with the three step model predicting the respective cutoff at the harmonic order of about (*IP* + 3.17 ⋅ *U*_*p*_)/*ω* ≈ 45 (Lewenstein et al., 1994), where *IP*, *U*_*p*_, and *ω* are the respective ionization potential, ponderomotive potential, and carrier frequency.

As the next step, we apply the 2FC approximation and allow the second 2*s*_+_ electron to be excited, first only in the 2*p*_+_ orbital (note that this approximation is analogous to using the minimal {1*s*, 2*s*, 2*p*} MCHF basis set). The respective HHG spectrum is shown in the second-from-the-bottom row of Figure 1 by a red curve. For a better comparison, the spectrum obtained in the previous SAE approximation is plotted in gray (note that this style of comparison of the result obtained in the two successive approximations will be kept thereafter, if not stated otherwise). As one can see, inclusion of the 2*p*_+_ orbital results in a decrease of the intensity of harmonics higher than about *k* > 15 by one to two orders of magnitude. Nevertheless, one can already see an appearance of harmonics beyond the SAE-cutoff up to about an order of 70. The middle panel of Figure 1 depicts the HHG spectrum computed in the 2FC approximation by additionally extending the ionic basis set with 3*s*_+_ and 4*s*_+_ orbitals. By comparing this spectrum (blue curve) with that obtained in the previous approximation (also shown in this panel), we observe an additional loss of the harmonic intensity in the range of 15 < *k* < 50.

Next, we allow the $1s_{+}^{2}$ electrons to be excited and ionized (2DC approximation). The respective HHG spectrum is plotted in the second-from-the-top row of Figure 1 against the previous result. A dramatic increase of intensity by a few orders of magnitude at almost all harmonic orders can be recognized. In addition, the computed spectrum exhibits now harmonics up to an order of about 100. Allowing in addition the $2s_{+}^{2}$ shell to relax does not significantly change the computed HHG spectrum (see the uppermost panel of Figure 1). An additional comparison of the spectrum computed in this approximation with that from the SAE calculations is given in the second-from-the-bottom row of Figure 2. While allowing the second 2*s*_+_ electron to be active significantly decreases the intensity of the computed HHG spectrum, ionization of the $1s_{+}^{2}$ shell recovers the intensity of harmonics to that computed in the SAE approximation. In addition, a prominent increase of intensity of 15 < *k* < 25 harmonics and an appearance of prominent 60 < *k* < 100 harmonics beyond the former cutoff can be recognized.

At the following step, we allow for the three-electron dynamics and correlations. The HHG spectrum computed in the 1FC approximation is compared to that obtained in the most complete 2DC approximation in the middle row of Figure 2. One can observe a slight attenuation of lower 15 < *k* < 25 and an amplification of higher *k* > 50 harmonic. Finally, the two uppermost rows of Figure 2 represent calculations obtained in the 2DC approximation by including all ionic orbitals with the principal quantum number *n* = 3 (the second-from-the-top row) and subsequently with *n* = 4 (the top row). The latter results employ the most complete basis set of ionic orbitals, but neglect the effect of three-electron dynamics and correlation (as indicated by the long vertical arrow in orange colour). As one can see, extension of the one-particle basis set further influences intensities of all harmonics with *k* > 15, but, importantly, it does not alter the extension of HHG spectrum up to an order of about 100, caused by allowing 1*s* ^2^ ionization and excitation.

As the last point of our study, we ensure that the effect observed here is independent of the basis set {*ϕ*_*α*_} of the bound orbitals. For this purpose, we perform analogous calculations employing the MCHF basis set described above. The results of those calculations (black curves) are compared in Figure 3 with the respective results from the ionic basis (red curves). In each step of subsequently improving approximations (note that SAE calculations are not possible in the MCHF basis set), the HHG spectra computed with the two chosen basis sets agree well with each other in overall. We thus conclude that the individual impacts of different physical processes, discussed above on the example of the ionic basis set, persist also in the MCHF basis set.

## Conclusion

Generation of high-order harmonics in the beryllium atom exposed to an intense linearly polarized 1850 nm laser pulse is studied beyond the single-active-electron approximation by the time-dependent restricted-active-space configuration-interaction method. In the calculations, we allowed only one of the electrons to be ionized and kept the other three electrons always bound to the nucleus, thereby neglecting the double-ionization process. This photoelectron was described in time-dependent wave packets with angular momenta *ℓ* ≤ 50. The dynamics of the remaining bound electrons was described by a set of discrete orbitals obtained either via Hartree-Fock or multiconfigurational Hartree-Fock methods in ionic or neutral beryllium, respectively. The active space of included configurations was systematically improved by allowing either specific basis states or specific types of configurations (or both) to be included. As soon as dynamics and correlations of the bound electrons are enabled, the computed spectrum of HHG exhibits considerably more harmonics of a higher order (as compared to that obtained in the SAE approximation). The richness of generated harmonics systematically increases with increasing the level of accuracy of the calculations. In particular, the HHG spectra computed in the most accurate approximations exhibit harmonics of up to an order of 100, and that in the SAE approximation only up to 60. Among the other included effects, excitation and ionization of the doubly-occupied 1*s* shell causes the most prominent effect on the computed spectrum of HHG. The presently obtained results are found to be independent of the basis set representing bound orbitals. As demonstrated in our previous work on HHG in He atom (Artemyev et al., 2017a), the effect of electron dynamics and correlations is also independent of the time envelope of employed laser pulses.

## Data Availability

The original contributions presented in the study are included in the article/Supplementary Material, further inquiries can be directed to the corresponding author.
